# Annotations

**Published:** 1897-09-04

**Authors:** 


### ANNOTATIONS.

### An Encouragement to Vaccination.

AN outbreak of small-pox has occurred in Glasgow, and the authorities are on the alert to prevent it spreading. They are doing their best to induce people to be re-vaccinated; but, as may well be believed, free vaccination is not inducement enough to the very people whose way of living makes them most liable to contract the disease. With those of the less cleanly classes who live in their own homes they can do little, but they have hit on an ingenious method of bribing the unvaccinated in common lodging-houses. The Corporation of Glasgow owns a number of lodging-houses itself, and there the sanitary official is offering free lodging for a week to everyone who will submit to be re-vaccinated; while in lodging-houses under private management it is willing to pay the proprietor two shillings and fourpence, the cost of a week's free lodging for the patient, for everyone who will submit to the operation. The bribe is proving successful; tramps, casuals, and vagabonds are being vaccinated in scores. Indeed, it is proving almost too successful, and the utmost vigilance has to be exercised by the authorities in order to prevent these people, by shifting about from one lodging house to another, from earning a steady livelihood by submitting to re-vaccination. The very original means taken to prevent the spread of the epidemic is costing Glasgow something, no doubt, but less than a widespread epidemic of small-pox—less in money in the end, and how much less in human lives!

### Oils and Explosions.

THE *Star* has republished, with the sensational title of "The Deadly 73°; a Business Tragedy in Two Hemispheres," some articles which have appeared in its columns. It must be owned that the subject matter justifies sensational treatment, for the booklet deals with the increasing number of fires and loss of life which can be traced to the explosion of oil lamps. In 1866 the number of such fires in London was 19; in 1895 it was 475. The cause of this increase is asserted by the author—and he quotes such high scientific authority as Lord Kelvin, Sir Henry Roscoe, Professor Ramsay, Professor Attfield, and Dr. Stevenson Macadam in support of his view—to be the low flash-point of the oil used by the poor. The "flash-point" of oil is the temperature at which oil will give off vapour; that is, the point at which a flame brought close to its surface will cause it to explode. In England the flash-point of oil is 73 deg. Fahr.; which means that anyone may lawfully sell oil which is liable to explode at a temperature of 73 deg. In Canada oil must not be used which has a lower flash-point than 85 deg.; in the State of Iowa, 105 deg.; and the English Government will have no oil used in its barracks that flashes at less than 100 deg., nor in its lighthouses that flashes at less than 120 deg. Yet there is infinitely less danger of a lamp being broken and oil spilled in barracks or lighthouse than in the living-room, which is usually the kitchen, of a poor man's house, with children playing, people moving to and fro—a fire at hand, and the temperature of the small, crowded apartment very often above 73 deg. It is in such circumstances that fires occur. Safer oils can be bought, but they are dearer; and how can the average poor purchaser know that the difference in price is the difference between safety and the chance of an agonising death? Up till 1879 the flash-point of the oil permitted to be sold in Britain was 100 deg.; but it was proved that, owing to inaccurate testing, oil of a lower degree of safety was often sold. Therefore, a more accurate tester was devised; but at the same time the flash-point was reduced to 73 deg., so that the one act nullified the other. The *Star* traced the increasing sale of these low-grade oils to the action of the Standard Oil Trust, which has its "tied-houses" all over Britain, after the fashion we associate with breweries, and sells here the oil, containing a heavy percentage of naphtha, with a low flash point, which cannot legally be sold in most of the American States. THE HOSPITAL has nothing to do with the methods of the Standard Oil Trust, nor the business morals of John D. Rockefeller, its head; it has no opinion to offer about monopolies, and it holds no brief for the Scottish mineral oil companies, the flash-point of whose oil is higher; but we think that there is strong evidence, supported by the statements of eminent scientists, that the present flash-point is too low, and that the limit should be fixed at a point which would give greater safety to those using oil-lamps than is the case at present.

### Bare Feet.

VISITORS to Scotland used to be horrified to see so many children running about barefooted. Bare feet are less common now than they were a generation ago, and perhaps the change, while showing a growing prosperity in the nation, is not altogether to be commended. Children's feet grow so fast that to keep them always properly shod is a matter that requires considerable care and some expenditure. It matters very little to a child's future well-being that at some period of its childhood the sleeves of a jacket have been too short or the skirt of a frock too scant; but the compression of feet in boots too tight or, even worse, too short, may be a cause of torment in future years. Infinitely better are bare feet than clumsy, heavy, ill-shapen boots. In the winter the feet may indeed want some protection from cold and wet, but during a great part of the year children may safely and healthfully go barefooted. Some mothers, by no means of the poorest class, are convinced that the comfort and symmetry of the feet in maturer years are largely to be gained by giving them freedom during the time of growth. At a very fashionable marriage some time ago a child bridesmaid was seen silk-robed, but shoeless. Where shoes to fit every stage of growth can easily be obtained, it may seem an excess of care, almost an affectation, to dispense with the conventional foot-covering; but if it makes it easier for the wife of a small tradesman—with whom the shoe problem is a difficult one, never solved in a comfortable or hygienic way—to let her children go barefoot if she sees the heir to a dukedom enjoying the full ease of his uncramped toes, we should beseech the duchess to take away his shoes. We have no doubt the young hope of the peerage would take his emancipation gladly. And if shoes are undesirable, how much more so are gloves. Except the thick woollen ones for winter warmth, gloves should be banished from a child's wardrobe. How many youngsters "dressed to death," or near it, would echo the complaint of a West Indian negro soldier when for the first time he donned full uniform: "Barracks for de feet bad 'nuff; barracks for d[illegible] hands too bad—too bad."

